# Denecimig (Mim8) prophylaxis over 52 weeks in adolescents and adults with hemophilia A: FRONTIER2 extension study

**DOI:** 10.1016/j.rpth.2026.106915

**Published:** 2026-08-27

**Authors:** Victor Jiménez-Yuste, Aby Abraham, Ciprian Tomuleasa, Hazza Alzahrani, Lize van Vulpen, Renchi Yang, Samya Obaji, Sanjay Ahuja, Sophie Susen, Steven R. Lentz, Amalie Roar Høgh, Ilgiz Rakhmatullin, Cédric Hermans

**Affiliations:** 1 Hospital Universitario La Paz (IdiPAZ), Universidad Autónoma de Madrid, Madrid, Spain; 2 Department of Clinical Haematology, Christian Medical College, Vellore, Tamil Nadu, India; 3 Hemostasis Lab, Department of Personalized Medicine and Rare Diseases, Medfuture Institute for Biomedical Research/Department of Hematology, Iuliu Hațieganu University of Medicine and Pharmacy, Cluj-Napoca, Romania; 4 Department of Hematology, King Faisal Specialist Hospital and Research Centre, Riyadh, Saudi Arabia; 5 Center for Benign Haematology, Thrombosis & Haemostasis, Van Creveldkliniek, University Medical Center Utrecht, Utrecht University, Utrecht, The Netherlands; 6 Thrombosis and Hemostasis Center, Institute of Hematology and Blood Diseases Hospital, Chinese Academy of Medical Sciences and Peking Union Medical College, Tianjin, China; 7 Cardiff Haemophilia Centre, University Hospital of Wales, Cardiff, UK; 8 Rainbow Babies and Children’s Hospital, Cleveland, Ohio, USA; 9 Hemostasis and Transfusion Department, University of Lille, Lille University Hospital, Lille, France; 10 Department of Internal Medicine, University of Iowa, Iowa City, Iowa, USA; 11 Novo Nordisk A/S, Søborg, Denmark; 12 Haemostasis and Thrombosis Unit, Division of Hematology, Cliniques Universitaires Saint-Luc, Université Catholique de Louvain (UCLouvain), Brussels, Belgium; 13 European Reference Network on Rare Hematological Diseases (ERN EuroBloodNet)

**Keywords:** activated FVIII mimetic, hemophilia A, inhibitors, patient-reported outcomes measures, prophylaxis

## Abstract

**Background:**

Denecimig (Mim8) is a fully human, activated factor VIII (FVIII) mimetic bispecific antibody in development for subcutaneous prophylaxis to prevent bleeding in persons with hemophilia A (HA).

**Objectives:**

This study aimed to report the efficacy and safety of denecimig over 52 weeks in adolescents and adults from FRONTIER2 (NCT05053139).

**Methods:**

Males and females (aged ≥12 years) with HA (any severity), with or without inhibitors, were enrolled. In the 26-week main phase, patients previously treated on-demand were randomized to continue on-demand treatment (arm 1) or receive denecimig injections once every week (QW; arm 2a) or once every month (QM; arm 2b); patients previously receiving clotting factor concentrate prophylaxis were randomized to receive denecimig QW (arm 3) or QM (arm 4). In the 26-week extension, patients in arm 1 switched to denecimig QW or QM; other arms followed the same dosing frequency. Annualized bleeding rate (ABR) of treated bleeds was estimated using a negative binomial model.

**Results:**

In the main phase, 281 patients were randomized; 272 entered the extension. Estimated mean ABR in the extension for arm 1 was 0.67 (QW) and 0.79 (QM). Mean ABR over 52 weeks was 0.37, 0.21, 2.02, and 1.65 in arms 2a, 2b, 3, and 4, respectively. Most baseline target joints resolved. No hypersensitivity reactions, thromboembolic events, or clinical evidence of neutralizing anti-denecimig antibodies occurred. Injection-site reactions occurred in 1.81% and 1.34% of patients administered denecimig QW and QM injections, respectively. Patient-reported physical function improved; treatment burden and joint pain was reduced.

**Conclusions:**

Denecimig maintained low ABRs over 52 weeks, was well tolerated, and improved patient-reported outcomes in adolescents and adults with HA with or without inhibitors.

## Essentials

• Denecimig (Mim8) is an activated factor VIII mimetic designed to prevent bleeds in hemophilia A.
• FRONTIER2 assessed denecimig prophylaxis over 52 weeks in adolescents/adults with hemophilia A.
• Denecimig maintained low bleeding rates and resolved the majority of baseline target joints.
• It improved patient-reported outcomes and was well tolerated, with few injection-site reactions.

## Graphical abstract

**Figure:**
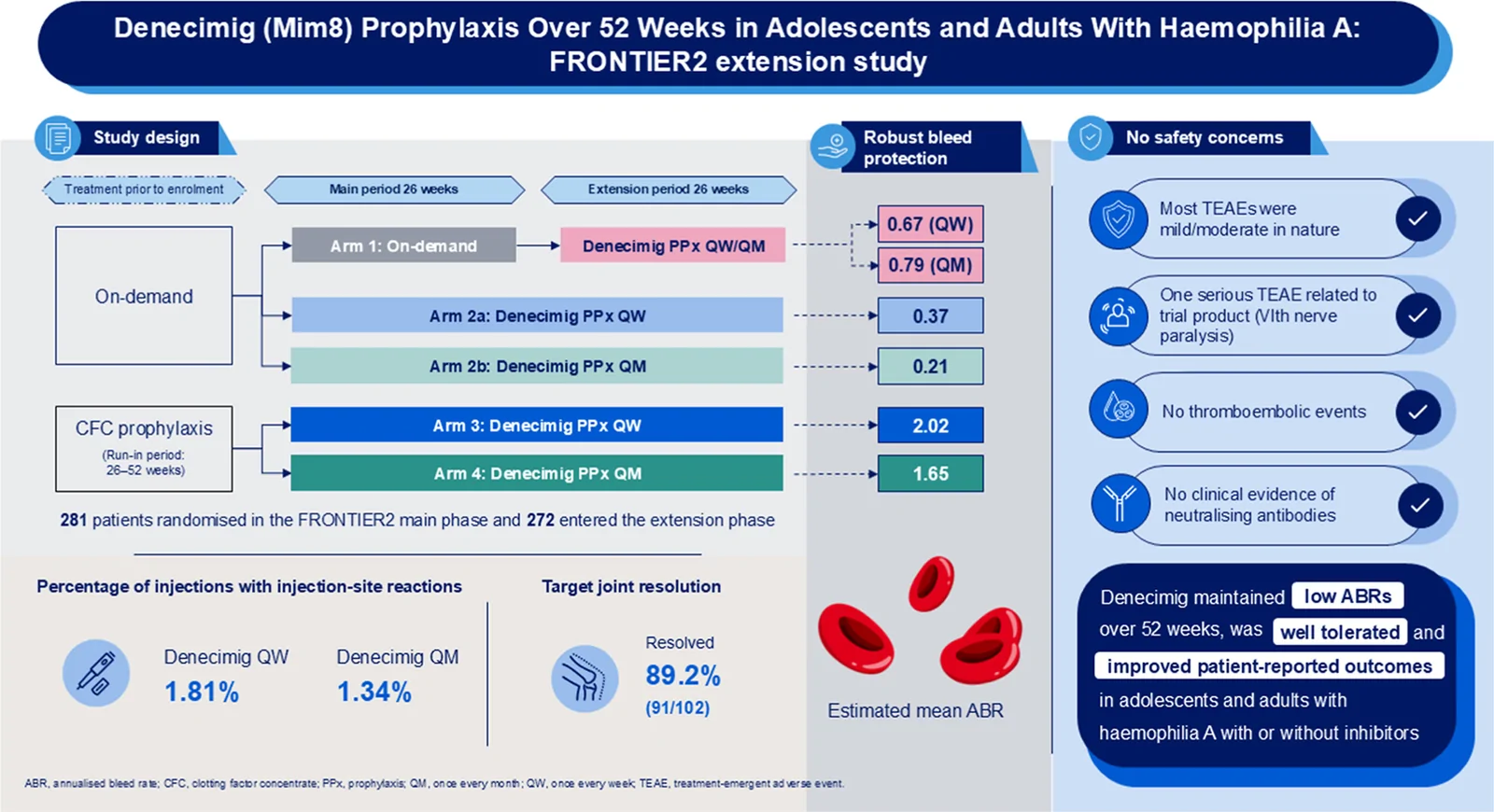

## 1 Introduction

Denecimig (Mim8) is a fully human, activated factor VIII (FVIIIa) mimetic bispecific antibody designed to prevent bleeding episodes and reduce treatment burden in persons with hemophilia A (HA) with or without inhibitors [1].

Contemporary options for HA treatment comprise FVIII replacement therapy and nonfactor therapies. FVIII replacement therapy requires frequent intravenous infusions and may be complicated by inhibitor development, which neutralizes efficacy [2]. Nonfactor therapies such as FVIIIa mimetics and rebalancing agents are designed to provide bleed protection in persons with HA with or without FVIII inhibitors [[3], [4], [5], [6]]. Emicizumab is a subcutaneous FVIIIa mimetic bispecific antibody that has demonstrated efficacy as a prophylactic treatment to prevent bleeds in persons with HA with or without inhibitors [5,6]. Although effective for bleed prevention, some patients continue to experience breakthrough bleeds during emicizumab prophylaxis [6,7]. Injection-site reactions (ISRs) reported with emicizumab, including erythema and pain, may negatively impair quality of life and can be particularly challenging in pediatric patients due to infusion volume [[6], [7], [8], [9], [10]]. Beyond bleeding outcomes, FVIIIa mimetics are designed to reduce treatment burden and therefore improve quality-of-life outcomes, as assessed by patient-reported outcomes (PROs) [[11], [12], [13], [14]].

Denecimig bridges activated factor IX (FIXa) and factor X (FX) to enhance thrombin generation [1]. It has been optimized for high cofactor activity to better mimic the function of endogenous FVIIIa [1]; the stimulatory effect of denecimig’s FIXa arm was enhanced to increase its ability to activate FX by >20,000-fold compared with FIXa alone [1]. In a first-in-human study, denecimig demonstrated ∼15-fold greater *ex vivo* potency than emicizumab in plasma from persons with HA [15].

The enhanced potency of denecimig allows sufficient thrombin generation with denecimig at low concentrations [1,16]. This facilitates administration with a fixed low injection volume of 0.8 mL, regardless of weight or dosing regimen, which minimizes the risk of potential dosing errors or drug wastage [1,16]. Denecimig uses a novel tiered dosing approach, starting with a one-time loading dose followed by fixed maintenance doses based on bodyweight range and preferred dosing frequency (once every week [QW], once every 2 weeks, or once every month [QM]) [16].

The FRONTIER clinical study program has been evaluating the efficacy and safety of denecimig for the treatment of HA with and without inhibitors. FRONTIER2 (ClinicalTrials.gov identifier NCT05053139) was a prospective, phase 3, open-label, controlled, randomized study in adolescents and adults. In the 26-week main phase, denecimig QW or QM significantly reduced annualized bleeding rate (ABR) of treated bleeds vs on-demand treatment and vs prior clotting factor concentrate (CFC) prophylaxis [17]. Denecimig was well tolerated, with no thromboembolic events, hypersensitivity reactions, or clinical evidence of neutralizing anti-denecimig antibodies observed [17].

Here, we report 52-week outcomes across the FRONTIER2 main and extension phase, designed to assess denecimig’s effect on bleed protection, longer-term safety, and PROs.

## 2 Methods

### 2.1 Study design and patients

FRONTIER2 investigated the efficacy of subcutaneous denecimig prophylaxis QW or QM in adolescents and adults (aged ≥12 years) with HA, with or without FVIII inhibitors. The FRONTIER2 study design has been described previously [17] (Supplementary Figure 1). Briefly, in the 26-week main phase, patients who had received on-demand treatment prior to the study (prestudy on-demand treatment group) were randomized 1:1:1 to continued on-demand treatment (arm 1) or to receive denecimig injections QW (arm 2a) or QM (arm 2b). Patients who had received CFC prophylaxis prior to the study (prestudy CFC prophylaxis group) completed a 26- to 52-week run-in period, followed by 1:1 randomization to denecimig QW (arm 3) or QM (arm 4). The main phase was followed by a 26-week extension; all patients who had received on-demand treatment in the main phase (arm 1) switched to denecimig QW or QM during the extension; denecimig dosing frequency was chosen based on patient preference in agreement with their investigator. All patients who had received denecimig QW or QM during the main phase (arms 2a, 2b, 3, and 4) continued the same dosing frequency during the extension.

This study included 27 patients from mainland China who were not included in the FRONTIER2 26-week primary analysis [17] because they had either not completed the main study period (*n* = 3, from arms 1, 2a, and 2b) at the time of the pivotal data cut or did not enter the run-in period and were therefore excluded from the primary endpoint analysis (*n* = 24, from arms 3 and 4). The inclusion and handling of the patient subgroup from mainland China was prespecified in the protocol in compliance with Chinese health authority requirements. Participants were enrolled under the same eligibility criteria and randomization procedures as all other study participants, minimizing the potential for selection bias.

Eligible participants were male or female (aged ≥12 years) individuals with HA (any severity), with or without inhibitors. During the 26 weeks before screening, participants in the prestudy on-demand treatment group were required to have ≥5 treated bleeds; participants in the prestudy CFC prophylaxis group with nonsevere HA (FVIII ≥1%) were required to have ≥1 treated bleed. Participants exposed to nonfactor prophylaxis within 6 months (or 5 half-lives, whichever was shorter) before randomization were excluded. The full inclusion and exclusion criteria have been reported previously [17].

FRONTIER2 was conducted at 100 sites across 32 countries. The study was approved by relevant Independent Ethics Committees and Institutional Review Boards. Written informed consent was obtained before study-related activities. There was no patient or public involvement in the design, conduct, and reporting of the trial.

### 2.2 Study intervention

Denecimig was administered subcutaneously with a fixed injection volume of 0.8 mL using a tiered dosing approach based on bodyweight (30 to <45 kg or ≥45 kg) and allocated dosing frequency (QW or QM) [17]. A one-time loading dose was administered to achieve steady state prior to maintenance dosing. Denecimig maintenance doses were fixed as 4 mg QW or 20 mg QM for participants weighing 30 to <45 kg and 9 mg QW or 46 mg QM for those weighing ≥45 kg.

For participants without FVIII inhibitors, treatment-requiring bleeding episodes were managed with FVIII products at the lowest dose expected to achieve hemostasis. For those with inhibitors, bleeding in a participant with a low-titer inhibitor (<5 Bethesda units) could be treated with FVIII concentrates in some instances. Recombinant activated factor VII (rFVIIa), at an initial dose of ≤90 μg/kg, was recommended as first-line treatment for bleeds requiring bypassing therapy. The use of activated prothrombin complex concentrate (PCC) and human coagulation FVIIa containing FX was only considered for severe bleeds if hemostatic control could not be achieved with rFVIIa, no other therapeutic options were available, and the clinical benefit was considered to outweigh the risk. Full details of breakthrough bleed management have been reported previously [17].

### 2.3 Study endpoints

The coprimary endpoints of FRONTIER2 at 26 weeks compared the number of treated bleeds during denecimig prophylaxis with continued on-demand treatment in the prestudy on-demand group, and compared the number of treated bleeds during denecimig prophylaxis with CFC prophylaxis during the run-in phase in the pre-study CFC prophylaxis group, data for which have been reported previously [17].

In the present study of 52-week outcomes, secondary endpoints from randomization to week 52 included occurrence of anti-denecimig antibodies and denecimig plasma concentration.

Additional assessments included the number of treated bleeds (ABR, referring to treated bleeds throughout this study), the number of treated bleeds by subtype (spontaneous, joint, traumatic, target joint), and target joint resolution. A target joint was defined as a joint in which 3 or more spontaneous bleeding episodes had occurred within 6 months before the date of the assessment; when there were ≤2 bleeds into the joint within a consecutive 12-month period, the joint was no longer considered a target joint (ie, resolved); 12 months of data were required to assess target joint resolution. Consumption of factor product per bleed, treatment-emergent adverse events (TEAEs), and the number of ISRs and laboratory assessments of coagulation parameters (D-dimer, prothrombin fragments 1 and 2, FIX antigen, FX antigen, fibrinogen) were additional assessments.

Changes in several PROs were also monitored over the 52-week study period as additional assessments. The Hemophilia Treatment Experience Measure (Hemo-TEM) is a validated, 26-item hemophilia-specific instrument for adolescents and adults [18] designed to assess treatment burden on a scale of 0 to 100; higher scores reflect greater burden, and a reduction of ≥8 points on the total score represents a clinically meaningful improvement in treatment burden.

The physical functioning domain of the validated Pediatric Quality of Life Inventory (PedsQL) was used to measure different aspects of activity, mobility, and energy on a scale of 0 to 100 [19]; higher scores indicate better physical functioning. Three versions of the PedsQL were used based on participant age at screening visit: PedsQL Teen (age 13-17 years), PedsQL Young Adult (age 18-25 years), PedsQL Adult (age 26+ years); patients used the same version of the PedsQL throughout the study.

The Joint Pain Rating Scale (JPRS) was used to assess joint pain severity on a scale of 0 to ‍10, with higher scores reflecting greater pain intensity. Patients responded to 2 items: (1) “In the past 7 days, how would you rate your worst pain in your joints?” and (2) “In the past 7 days, how much did joint pain interfere with your daily activities?”.

The Patient Global Impression of Change (PGI-C) measures were used to assess change in physical function and pain intensity. Patients were asked, “How would you rate your current ability to do every day physical activities compared to your ability to do these activities before the study?” to assess change in PGI-C physical function, with responses on a 5-item scale ranging from “Much worse” to “Much better.” Additionally, they were asked, “How much pain in your joints do you have now compared to before the study?” to assess change in PGI-C pain intensity, with responses on a 5-item scale ranging from “Much more pain” to “Much less pain.”

The Haemophilia Patient Preference Questionnaire (HPPQ) is a bespoke hemophilia questionnaire, not yet validated at the time of writing. The HPPQ was used to evaluate patients’ treatment preference, with patients asked, “Overall what treatment do you prefer?,” with response options being “Current,” “Previous,” and “No preference.” If “Current” or “Previous” was selected, a follow-up question was asked, “How strong is that preference?,” with patients responding “Very strong,” “Fairly strong,” or “Not very strong.”

### 2.4 Statistical analysis

The full analysis set used for efficacy evaluation included all randomized patients. Assessments relating to number of treated bleeds were carried out using a negative binomial regression model, which varied based on treatment arm. All arms used the logarithm of exposure time as offset. No analyses were performed to compare treatment arms. Objectives, endpoints, additional analyses, and estimands reported for the 27 additional patients from mainland China in the present analysis were the same as for the rest of the study population.

For arm 1, the model used treatment as factor and historical bleeding frequency (during the 12 months before enrolment) as covariate. For arms 2a and 2b, the model used treatment, inhibitor status, and historical bleeding frequency (ABR <18 or ABR ≥18) as factors and historical bleeding frequency (during the 12 months prior to enrollment) as covariate. For arms 3 and 4, the model used severity of hemophilia (<1% or ≥1% FVIII activity) as a factor, and within-participant repeated measurements were incorporated using an unstructured covariance structure. Further details regarding the negative binomial regression model for bleed subtypes can be found in the Supplementary Appendix.

All efficacy assessments other than treated bleeds were reported using descriptive statistics based on the full analysis set.

The safety analysis set used for safety evaluation included all patients randomized and exposed to the study product. Safety assessments were reported using descriptive statistics based on the safety analysis set.

Denecimig plasma concentrations from randomization to week 52 were quantified by immunoassay and analyzed against time data using a nonlinear mixed-effects pharmacokinetic model, quantifying covariate effects on denecimig exposure.

Further details regarding the statistical analysis plan and study protocol, including sample size determination, handling of intercurrent events, and multiplicity strategy, have been reported previously [17].

## 3 Results

### 3.1 Patient demographics and disposition

In total, 281 patients were randomized in the 26-week main phase (Supplementary Figure 2). Overall, baseline demographics were generally comparable across treatment arms, with the exception of inhibitor status. Mean (SD) age at baseline across treatment arms ranged from 30 (13) to 33 (16) years, with 11.1% to 19.0% of patients aged ≥50 years; the majority of participants had severe HA (63.6%-90.5%), were ≥18 years old (66.7%-76.2%) and were inhibitor negative (54.5%-98.2%; Table 1 [17]).

**Table 1:** Demographics and baseline characteristics.^a^

Characteristic	Prestudy on-demand treatment group			Prestudy CFC prophylaxis group	
	On-demand (arm 1)	Denecimig QW (arm 2a)	Denecimig QM (arm 2b)	Denecimig QW (arm 3)	Denecimig QM (arm 4)
	*n* = 18	*n* = 22	*n* = 21	*n* = 111	*n* = 109
Age					
Age at baseline, y, mean (SD)	30 (13)	31 (16)	33 (16)	31 (16)	31 (16)
12-17 y	6 (33.3)	6 (27.3)	5 (23.8)	37 (33.3)	33 (30.3)
≥18 y	12 (66.7)	16 (72.7)	16 (76.2)	74 (66.7)	76 (69.7)
≥50 y	2 (11.1)	3 (13.6)	4 (19.0)	18 (16.2)	13 (11.9)
Weight					
Weight, kg, mean (SD)	62.2 (16.3)	70.8 (21.1)	69.9 (18.5)	75.1 (20.5)	76.3 (20.0)
30 to <45 kg	2 (11.1)	2 (9.1)	4 (19.0)	7 (6.3)	2 (1.8)
≥45 kg	16 (88.9)	20 (90.9)	17 (81.0)	104 (93.7)	107 (98.2)
Sex					
Female	1 (5.6)	0	2 (9.5)	0	1 (0.9)
Male	17 (94.4)	22 (100.0)	19 (90.5)	111 (100)	108 (99.1)
Race					
Asian	12 (66.7)	12 (54.5)	12 (57.1)	25 (22.5)	28 (25.7)
Black or African American	1 (5.6)	1 (4.5)	2 (9.5)	1 (0.9)	2 (1.8)
Native Hawaiian or other Pacific Islander	0	0	0	1 (0.9)	0
White	4 (22.2)	9 (40.9)	7 (33.3)	72 (64.9)	68 (62.4)
Other	1 (5.6)	0	0	7 (6.3)	7 (6.4)
Missing	0	0	0	5 (4.5)	4 (3.7)
Current inhibitor status					
Positive	8 (44.4)	10 (45.5)	9 (42.9)	2 (1.8)	2 (1.8)
Negative	10 (55.6)	12 (54.5)	12 (57.1)	109 (98.2)	107 (98.2)
Hemophilia severity					
Mild	2 (11.1)	2 (9.1)	1 (4.8)	0	1 (0.9)
Moderate	2 (11.1)	6 (27.3)	1 (4.8)	14 (12.6)	15 (13.8)
Severe	14 (77.8)	14 (63.6)	19 (90.5)	97 (87.4)	93 (85.3)

Values are *n* (%) unless otherwise specified.

CFC, clotting factor concentrate; QM, once every month; QW, once every week.

a Study population included 27 patients from mainland China who were not included in the FRONTIER2 26-week primary analysis [17].

In total, 273 (97.2%) patients completed the main phase (total exposure time in the main phase: 137.8 patient-years) and 272 entered the extension. One patient withdrew from the study without continuing to the extension phase and chose to be treated with their previously used medication (Supplementary Figure 2). Patients previously treated on-demand in the main phase (arm 1; *n* = 18) switched to denecimig QW (*n* = 8) or denecimig QM (*n* = 10) during the extension. The remaining patients continued to receive their same dosing frequency of denecimig QW (arm 2a, *n* = 21; Arm 3, *n* = 104) or denecimig QM (arm 2b, *n* = 21; arm 4, *n* = 108); 270 (99.3%) patients completed the extension phase (total exposure time in the extension phase: 138.5 years) (Supplementary Figure 2).

### 3.2 Efficacy

In arm 1, estimated mean ABR with continued on-demand treatment was 16.09 at week 26 (Figure 1; Table 2); following transition to denecimig, the estimated mean ABR during the 26-week extension was 0.67 with denecimig QW and 0.79 with denecimig QM; the median ABR was 0.00 for both dosing frequencies. In the prestudy on-demand group initially randomized to denecimig (arm 2), the estimated mean ABR over 52 weeks was 0.37 with denecimig QW (arm 2a) and 0.21 with denecimig QM (arm 2b); the median ABR was 0.00 for both dosing frequencies. In the prestudy CFC prophylaxis group, the estimated mean ABR over 52 weeks was 2.02 with denecimig QW (arm 3) and 1.65 with denecimig QM (arm 4); the median ABR was 0.00 and 0.95, respectively (Figure 1; Table 2). Estimated mean ABR was similar between the main and extension phases for the prestudy on-demand group randomized to denecimig (arms 2a and 2b). Estimated mean ABR showed a numerical improvement in the extension phase compared with the main phase in the prestudy CFC prophylaxis group randomized to denecimig (arms 3 and 4) (Supplementary Tables 1 and 2).

**Figure 1:**
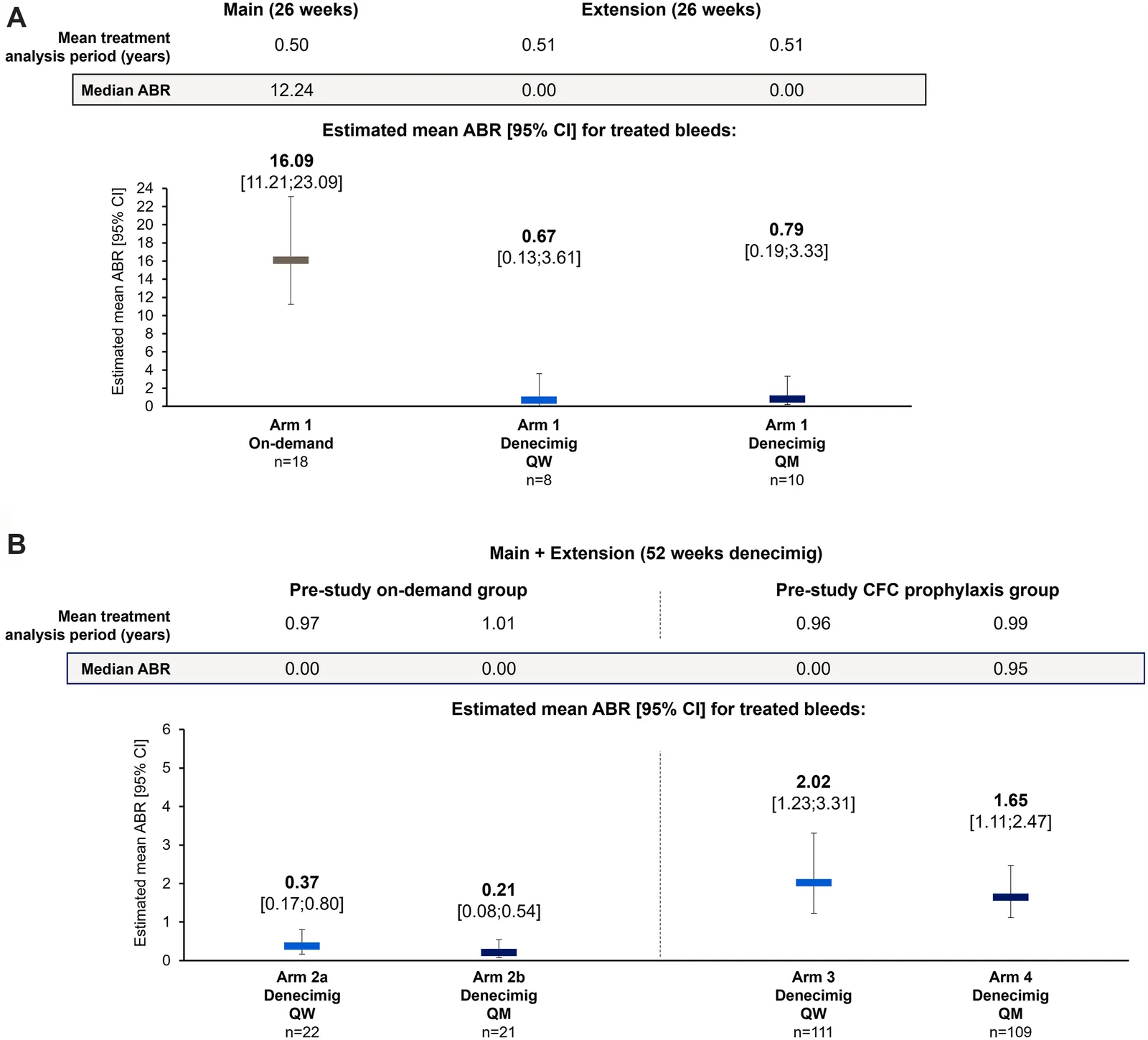
Annualized bleeding rate with denecimig prophylaxis in (A) patients who transitioned from on-demand treatment to denecimig at week 26 and (B) patients in the prestudy on-demand treatment group and prestudy CFC prophylaxis group. (A) Arm 1 (patients receiving continued on-demand treatment during the 26-week main phase and transitioning to denecimig QW or QM during the extension). The negative binomial model for the main phase included treatment, inhibitor status, and historical bleed frequency (ABR <18 or ABR ≥18) as factors, historical bleed frequency as covariate, and logarithm of treatment duration as offset. The negative binomial model for the extension phase included treatment as factor, historical bleed frequency as covariate, and logarithm of treatment duration as offset. (B) Prestudy on-demand treatment group (denecimig QW and QM; arms 2a and 2b): negative binomial model includes treatment, inhibitor status, and historical bleed frequency (ABR <18 or ABR ≥18) as factors, historical bleed frequency as covariate, and logarithm of treatment duration as offset. Prestudy CFC prophylaxis group (denecimig QW and QM; arms 3 and 4): negative binomial model includes treatment and hemophilia severity (≥1% or <1% factor VIII) as factors, logarithm of treatment duration as offset, and within-participant repeated measurements incorporated using an unstructured covariance structure. ABR, annualized bleeding rate; CFC, clotting factor concentrate; QM, once every month; QW, once every week.

**Table 2:** Treated bleeding episodes during the main and extension phases of FRONTIER2.

Bleeding episode characteristic	Prestudy on-demand treatment group				Prestudy CFC prophylaxis group	
	Denecimig QW (arm 1)	Denecimig QM (arm 1)	Denecimig QW (arm 2a)	Denecimig QM (arm 2b)	Denecimig QW (arm 3)	Denecimig QM (arm 4)
	extension	extension	main and extension	main and extension	main and extension	main and extension
	*n* = 8	*n* = 10	*n* = 22	*n* = 21	*n* = 111	*n* = 109
Mean exposure time, y	0.51	0.51	0.97	1.01	0.96	0.99
Number of patients with bleeds, *n* (%)	1 (12.5)	3 (30.0)	3 (13.6)	3 (14.3)	55 (49.5)	56 (51.4)
Number of patients with zero bleeds, *n* (%)	7 (87.5)	7 (70.0)	19 (86.4)	18 (85.7)	56 (50.5)	53 (48.6)
Number of treated bleeds	3	4	13	8	177	181
Cause of bleed, *n* (%)						
Spontaneous	3 (100.0)	1 (25.0)	2 (15.4)	7 (87.5)	93 (52.5)	86 (47.5)
Traumatic	0 (0.0)	3 (75.0)	11 (84.6)	1 (12.5)	78 (44.1)	85 (47.0)
Surgical^a^	0	0	0	0	6 (3.4)	10 (5.5)
Location of bleed, *n* (%)						
Joint	2 (66.7)	1 (25.0)	11 (84.6)	2 (25.0)	105 (59.3)	116 (64.1)
Spontaneous bleeds located in joints^b^	2 (66.7)	0	2 (100.0)	1 (14.3)	63 (67.7)	71 (82.6)
Classification of bleed, *n* (%)						
Mild/moderate	3 (100)	4 (100)	13 (100)	8 (100)	174 (98.3)	179 (98.9)
Severe bleeds^c^	0	0	0	0	3 (1.7)	2 (1.1)
ABR						
Estimated mean [95% CI]	0.67 [0.13; 3.61]	0.79 [0.19; 3.33]	0.37 [0.17; 0.80]	0.21 [0.08; 0.54]	2.02 [1.23; 3.31]	1.65 [1.11; 2.47]
Median [IQR]	0.00 [0.00; 0.00]	0.00 [0.00; 1.83]	0.00 [0.00; 0.00]	0.00 [0.00; 0.00]	0.00 [0.00; 1.97]	0.95 [0.00; 1.85]

ABR was estimated using a negative binomial regression model.

ABR, annualized bleeding rate; CFC, clotting factor concentrate; QM, once every month; QW, once every week.

a In arm 3, 5 of the surgical bleeds were located in the mouth/nose/gums, and 1 was reported as “other”; in arm 4, 2 of the surgical bleeds were located in the gastrointestinal system, and 8 were located in the mouth/nose/gums.

b Percentage values are the proportion of the total number of spontaneous bleeds observed.

c Severe bleeds included: intracranial, retroperitoneal, iliopsoas, and internal neck bleeds; muscle bleeds with compartment syndrome; bleeds associated with a significant decrease in hemoglobin level (>3 g/dL); bleeding episodes that require hospitalization; and all life-threatening bleeding episodes.

One patient in arm 3 who received denecimig QW for 108 days reported 22 bleeding events. All 22 events were considered by the investigator to be spontaneous and mild-to-moderate in severity; 19 events were in one joint (ankle), and the patient had no anti-denecimig antibodies. In a post hoc sensitivity analysis of denecimig QW prophylaxis that excluded this patient, the estimated mean ABR over 52 weeks decreased to 1.53 (95% CI, 1.12-2.11) compared with the primary analysis (outlier included) with an estimated mean ABR of 2.02 (95% CI, 1.23-3.31).

In the prestudy on-demand treatment group (arms 2a and 2b), estimated mean ABR with denecimig was numerically higher for inhibitor-negative patients (0.58; 95% CI, 0.30-1.10; *n* = 24) compared with inhibitor-positive (0.09; 95% CI, 0.02-0.39; *n* = 19) patients. In the prestudy CFC prophylaxis group, estimated mean ABR was comparable between participants with severe (1.66; 95% CI, 1.28-2.16]; *n* = 190) and nonsevere (1.47; 95% CI, 0.78-2.79]; *n* = 30) hemophilia (Supplementary Tables 3 and 4).

In the 26-week main phase of the study, the proportion of patients with zero treated bleeds was 86.4% with denecimig QW (arm 2a), 90.5% with denecimig QM (arm 2b), 66.7% with denecimig QW (arm 3), and 63.3% with denecimig QM (arm 4; Figure 2). The proportion of patients with zero bleeds was similar between the main phase and extension phase of the study (Figure 2), with the proportion of patients with zero bleeds increasing slightly in arms 2a, 3, and 4 during the extension phase. In the 26-week extension, the proportion of patients with zero treated bleeds was 87.5% with denecimig QW (arm 1), 70.0% with denecimig QM (arm 1), 90.5% with denecimig QW (arm 2a), 85.7% with denecimig QM (arm 2b), 70.2% with denecimig QW (arm 3), and 68.5% with denecimig QM (arm 4). Across the 52-week treatment period, zero bleeds were experienced by 86.4% (QW, arm 2a), 85.7% (QM, arm 2b), 50.5% (QW, arm 3) and 48.6% (QM, arm 4; Table 2) of patients.

**Figure 2:**
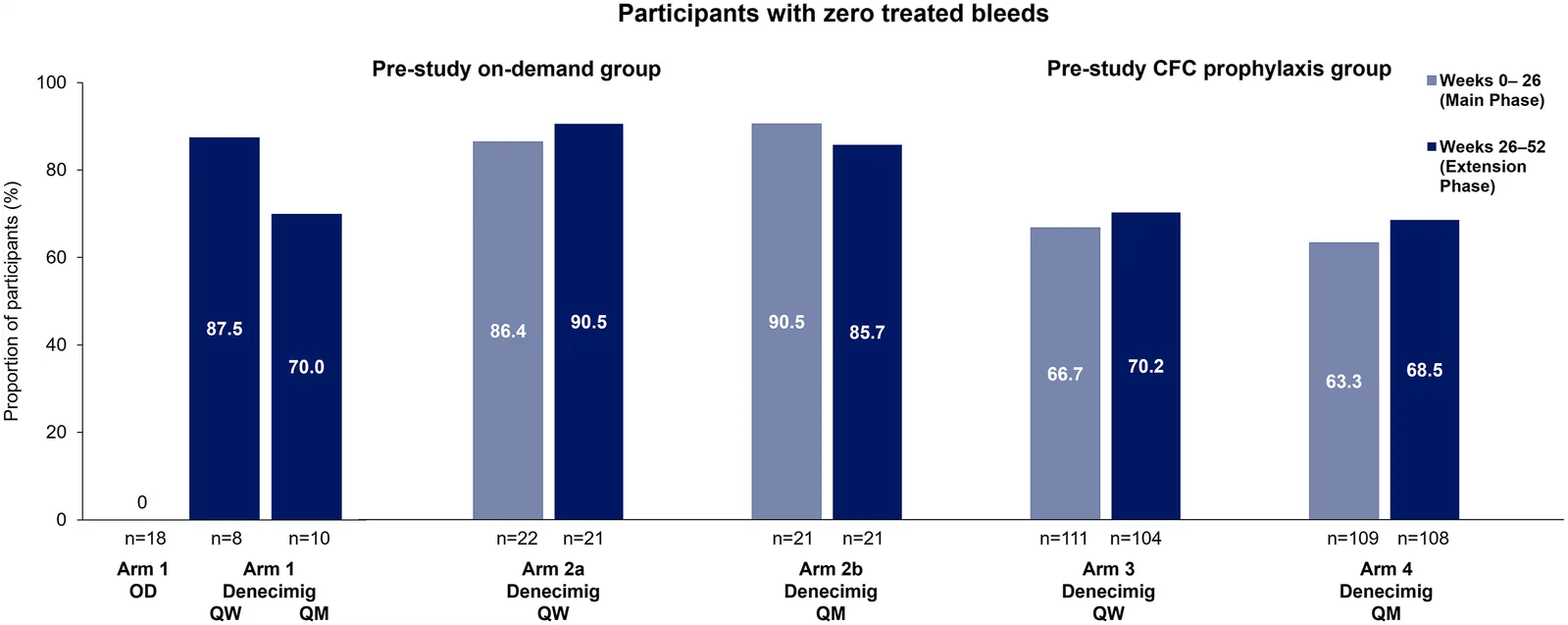
Proportion of patients with zero treated bleeds by treatment phase. CFC, clotting factor concentrate; OD, on-demand; QM, once every month; QW, once every week.

In arm 1, there were 3 (denecimig QW) and 4 (denecimig QM) patients who reported treated bleeds during denecimig prophylaxis in the extension period; all bleeds were mild/moderate in severity (Table 2). In the prestudy on-demand group of patients who received denecimig QW (arm 2a) or QM (arm 2b) for 52 weeks, 13 and 8 treated bleeds were reported, respectively; all were mild/moderate. In the prestudy CFC prophylaxis group of patients who received denecimig QW (arm 3) or QM (arm 4) for 52 weeks, 177 and 181 treated bleeds were reported, respectively; the majority (98.3% and 98.9%) were mild/moderate (Table 2). There were no clear differences in spontaneous bleeds or joint bleeds between QW and QM dosing (Table 2). Bleeding outcomes by subtype of bleed are reported in Supplementary Table 5.

During denecimig prophylaxis in the main and extension phases, the majority of bleeds in each treatment arm were treated with 1 injection (Supplementary Table 6). FVIII (including standard recombinant factor concentrate and extended half-life factor concentrate) was frequently used to manage bleeding episodes (Supplementary Table 6); only 1 patient (denecimig QM; arm 2b) used activated PCC/PCC to treat bleeds (dose: 14.5 IU/kg); 1 patient in each of the denecimig QW arms (arm 1, arm 2a, and arm 3) used a bypassing agent (rFVIIa or FVIIa + FX) to treat bleeds.

The majority of patients with target joints at baseline experienced resolution of all target joints after 52 weeks of denecimig treatment (Supplementary Table 7). Overall, 23 of 26, 28 of 28, 21 of 24, and 19 of 24 baseline target joints were resolved during denecimig prophylaxis in arms 2a, 2b, 3, and 4, respectively.

### 3.3 Pharmacokinetics

Mean denecimig concentrations were stable and comparable within QW and QM dosing frequencies (Figure 3). Mean (SD) denecimig concentrations at week 52 were 5.23 (2.02), 4.93 (1.96), and 4.81 (1.65) μg/mL for QW (arm 1, arm 2a, and arm 3, respectively) and 3.93 (1.54), 3.50 (1.42), and 4.18 (1.76) μg/mL for QM (arm 1, arm 2b, and arm 4).

**Figure 3:**
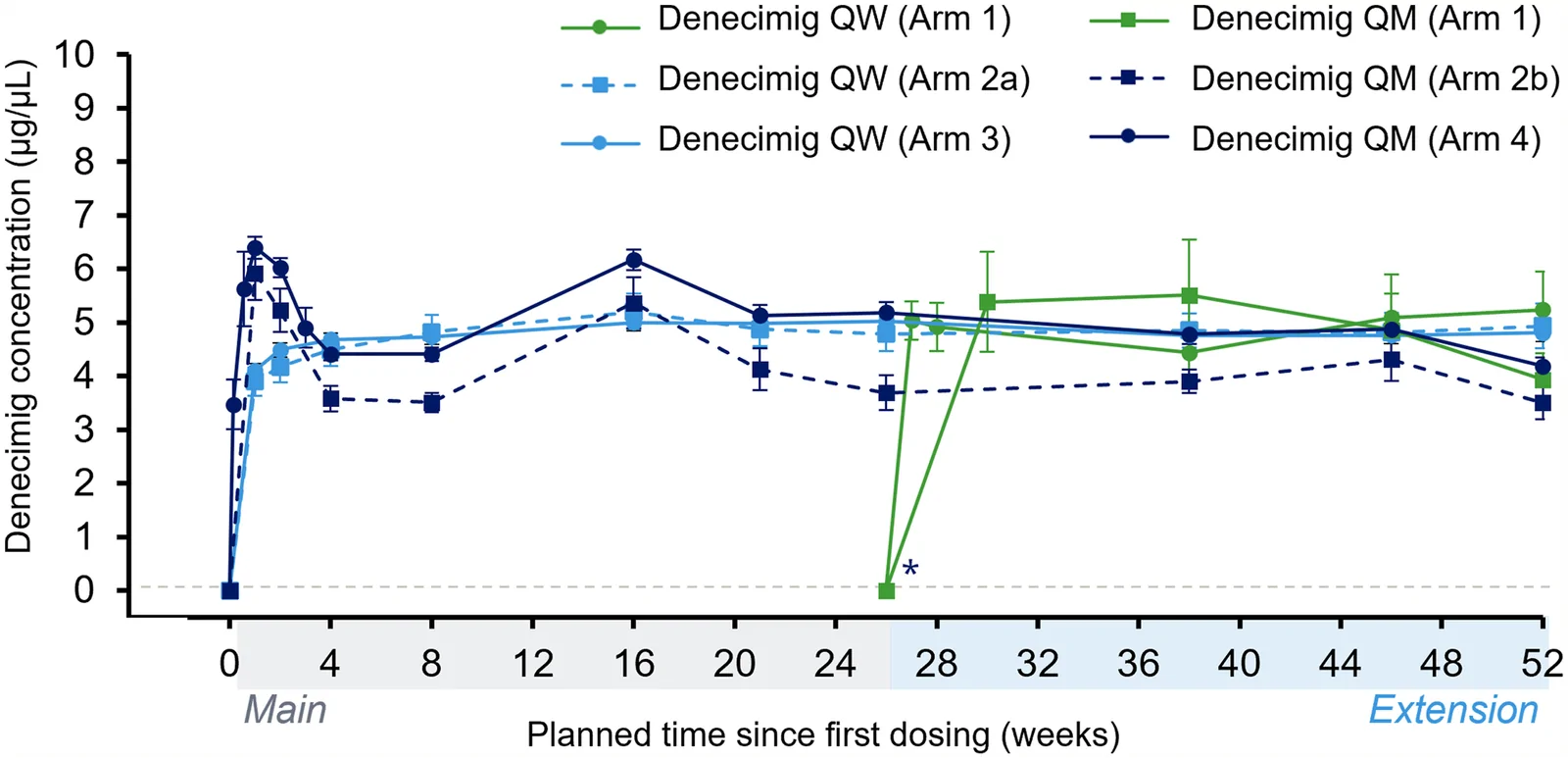
Mean of denecimig plasma concentration (linear scale). ∗Patients who received on-demand treatment during the 26-week main phase (arm 1) transitioned to denecimig prophylaxis (QW or QM) upon entry into the extension phase at week 26. Grey dashed line represents lower limit of quantification; error bars represent SEM. Peak concentrations at week 16 were collected postdose (approximately 3 days after QW dosing and 10 days after QM dosing). Samples at other time points were collected predose, unless otherwise specified in the study protocol. QM, once every month; QW, once every week.

### 3.4 Safety

For patients receiving denecimig QW (*n* = 141; arm 1 QW, arm 2a, and arm 3), TEAEs were reported in 104 (73.8%) patients, serious TEAEs in 8 (5.7%) patients, and TEAEs leading to discontinuation in 3 (2.1%) patients; the majority of TEAEs were mild/moderate in severity. For patients receiving denecimig QM (*n* = 140; arm 1 QM, arm 2b, and arm 4), TEAEs were reported in 100 (71.4%) patients, serious TEAEs in 9 (6.4%) patients, and TEAEs leading to discontinuation in 1 (0.7%) patient; the majority of TEAEs were mild/moderate in severity. Across all treatment arms, no TEAEs with fatal outcome, no hypersensitivity reactions, and no TEAEs of special interest (thromboembolic events or thrombotic microangiopathies) were reported (Table 3; Supplementary Tables 8 and 9).

**Table 3:** Number and type of treatment-emergent adverse events reported during the main and extension phases of FRONTIER2.

TEAE characteristic	Pre-study on-demand treatment group				Pre-study CFC prophylaxis group	
	Denecimig QW (arm 1)	Denecimig QM (arm 1)	Denecimig QW (arm 2a)	Denecimig QM (arm 2b)	Denecimig QW (arm 3)	Denecimig QM (arm 4)
	extension	extension	main and extension	main and extension	main and extension	main and extension
	*n* = 8	*n* = 10	*n* = 22	*n* = 21	*n* = 111	*n* = 109
Total exposure time, y	4.09	5.17	21.65	21.23	109.95	109.85
All TEAEs, *n* (%) E [R]	4 (50.0) 9 [2.20]	4 (40.0) 6 [1.16]	11 (50.0) 30 [1.39]	8 (38.1) 34 [1.60]	89 (80.2) 436 [3.97]	88 (80.7) 350 [3.19]
Serious TEAEs, *n* (%) E [R]	0	0	1 (4.5) 2 [0.09]	0	7 (6.3) 9 [0.08]	9 (8.3) 12 [0.11]
TEAEs of special interest (thromboembolic events), *n*	0	0	0	0	0	0
TEAEs by severity, *n* (%) E [R]						
Mild	4 (50.0) 8 [1.96]	3 (30.0) 5 [0.97]	8 (36.4) 22 [1.02]	8 (38.1) 34 [1.60]	85 (76.6) 369 [3.36]	82 (75.2) 282 [2.57]
Moderate	1 (12.5) 1 [0.24]	1 (10.0) 1 [0.19]	4 (18.2) 6 [0.28]	0	33 (29.7) 60 [0.55]	37 (33.9) 64 [0.58]
Severe	0	0	2 (9.1) 2 [0.09]	0	5 (4.5) 7 [0.06]	4 (3.7) 4 [0.04]
TEAEs related to denecimig treatment^a^, *n* (%) E [R]	2 (25.0) 3 [0.73]	0	4 (18.2) 5 [0.23]	2 (9.5) 3 [0.14]	32 (28.8) 152 [1.38]	22 (20.2) 42 [0.38]
TEAEs leading to permanent discontinuation of study product^b^, *n* (%) E [R]	0	0	1 (4.5) 1 [0.05]	0	2 (1.8) 3 [0.03]	1 (0.9) 1 [0.01]
TEAEs with additional data collection category, *n* (%) E [R]						
Injection-site reactions	1 (12.5) 1 [0.24]	0	2 (9.1) 2 [0.09]	1 (4.8) 2 [0.09]	14 (12.6) 124 [1.13]	11 (10.1) 22 [0.20]
Hypersensitivity reactions	0	0	0	0	0	0
Medication errors, and misuse and abuse^c^	0	0	0	0	1 (0.9) 1 [0.01]	0

CFC, clotting factor concentrate; E, number of adverse events; QM, once every month; QW, once every week; R, rate (number of events per patient-year of exposure); TEAE, treatment-emergent adverse event.

a Investigator’s categorization following clinical study protocol.

b 1 participant (denecimig QW; arm 2a) discontinued treatment due to a serious TEAE with probable relation to trial product (sixth cranial nerve paralysis); 1 participant (denecimig QW; arm 3) discontinued treatment due to 2 nonserious TEAEs with possible relation to trial product (sleep disorder and dyspepsia); 2 patients (arms 3 and 4) discontinued treatment due to a nonserious and a serious TEAE, respectively, with unlikely relation to trial product (coronary artery disease and synovitis).

c The participant administered 3 doses within 1 dosing period.

ISRs were reported in 12.1% of patients receiving denecimig QW (arm 1 QW, arm 2a, and arm 3), and in 8.6% of patients receiving denecimig QM (arm 1 QM, arm 2b, and arm 4). ISRs were observed in 1.81% (*n* = 127 of 7013) of injections with QW and 1.34% (*n* = 24 of 1789) of injections with QM (Supplementary Table 10). The most commonly reported signs/symptoms included erythema and pruritus (itching), and 2 of the ISRs included burning or pain as a symptom. The majority of ISRs were transient; all were mild in severity, and all recovered/resolved.

Anti-denecimig antibodies were detected in 7.5% (*n* = 21 of 281) of patients during denecimig prophylaxis (Supplementary Table 11). All antibodies were low or medium titer; no clinical evidence of neutralizing effect was noted, based on preserved denecimig concentration and activity, activated partial thromboplastin time within reference range, and no impact on bleeding patterns.

There were no clinically relevant findings in laboratory assessments, including coagulation parameters (D-dimer, prothrombin fragments 1 and 2, FIX antigen, FX antigen, fibrinogen; Supplementary Figure 3).

### 3.5 Patient-reported outcomes

Comparable PRO results were observed across QW and QM dosing; therefore, results were pooled by dosing frequency. In arm 1, the mean change from week 26 to week 52 in Hemo-TEM total score was –16.0 (denecimig QW/QM; extension period only; Figure 4 and Supplementary Table 12), indicating a decreased treatment burden. Mean change from baseline to week 52 in Hemo-TEM total score was –12.7 in the prestudy on-demand treatment group (denecimig QW/QM; arms 2a and 2b) and –9.7 in the prestudy CFC prophylaxis group (denecimig QW/QM; arms 3 and 4). A clinically meaningful ≥8-point reduction was achieved by 61.5% (denecimig QW/QM; arm 1), 55.2% (denecimig QW/QM; arms 2a and 2b), and 46.2% (denecimig QW/QM; arms 3 and 4) of patients.

**Figure 4:**
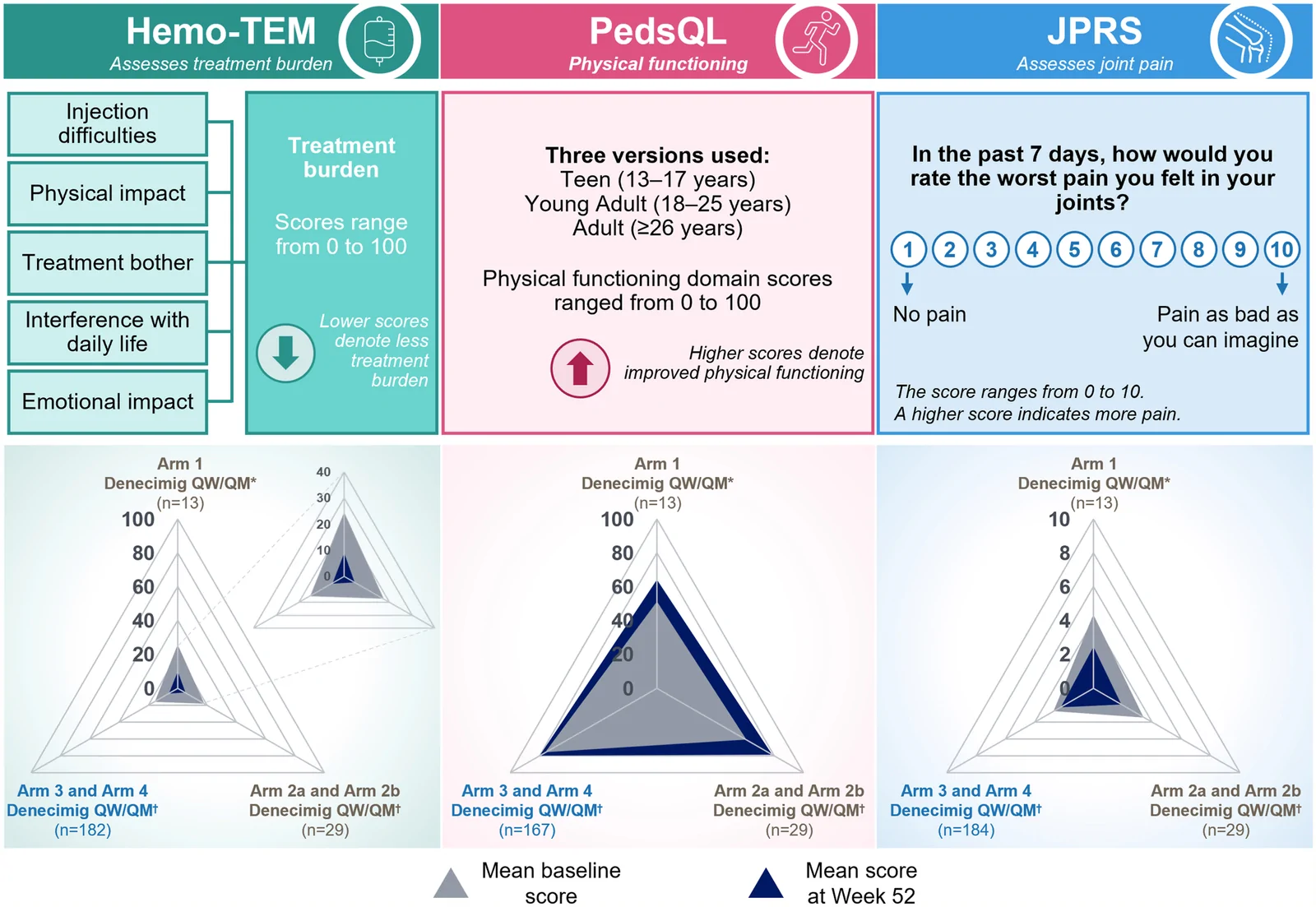
Change from baseline to week 52 in Hemo-TEM, PedsQL, and JPRS. Hemo-TEM graph includes a view with a reduced scale to visualize the difference between baseline and week 52 more clearly. ∗During the study, patients received 26 weeks of on-demand treatment and 26 weeks of denecimig; baseline score is from start of denecimig treatment. ^†^During the study, patients received 52 weeks of denecimig. Hemo-TEM, Hemophilia Treatment Experience Measure; JPRS, Joint Pain Rating Scale; PedsQL, Pediatric Quality of Life Inventory; QM, once every month; QW, once every week.

In arm 1 (denecimig QW/QM), the mean change from week 26 to week 52 in PedsQL physical functioning score was +13.0, indicating an improvement in physical functioning. The mean change from baseline to week 52 was +17.6 (denecimig QW/QM; arms 2a and 2b) and +4.0 (denecimig QW/QM; arms 3 and 4) (Figure 4 and Supplementary Table 10).

In arm 1 (denecimig QW/QM), the mean change from week 26 to week 52 in joint pain, as assessed by the JPRS score, was –1.8 points, indicating a reduction in joint pain. Mean change from baseline to week 52 was –1.5 points (denecimig QW/QM; arms 2a and 2b) and –0.4 points (denecimig QW/QM; arms 3 and 4) (Figure 4 and Supplementary Table 10).

At week 52, 58.8% (denecimig QW/QM; arm 1), 65.0% (denecimig QW/QM; arms 2a and 2b), and 40.1% (denecimig QW/QM; arms 3 and 4) rated their physical activity as “Much better” with the PGI-C physical function measure compared with before the study (Supplementary Table 10). Using the PGI-C pain intensity score, 64.7% (denecimig QW/QM; arm 1), 75.0% (denecimig QW/QM; arms 2a and 2b), and 37.5% (denecimig QW/QM; arms 3 and 4) reported “Much less pain” in their joints at week 52 compared with before the study (Supplementary Table 10).

At week 52, the majority of patients reported preference for denecimig over previous treatment, as assessed by the HPPQ. A “Very strong” preference for denecimig compared with previous treatment was reported by 64.7% (denecimig QW/QM; arm 1), 65.0% (denecimig QW/QM; arms 2a and 2b), and 69.8% (denecimig QW/QM; arms 3 and 4) of patients (Supplementary Table 10).

## 4 Discussion

Over 52 weeks of the phase 3 FRONTIER2 study, denecimig prophylaxis demonstrated sustained bleed protection whereby low ABRs were maintained across the treatment period, the majority of patients experienced zero treated bleeds, and most baseline target joints were resolved. Denecimig was well tolerated, with no thromboembolic events, hypersensitivity, or clinical evidence of neutralizing antibodies observed, and a low proportion of ISRs. Denecimig prophylaxis was associated with improvements across the PROs assessed, including a reduction in treatment burden, improved physical function, decreased joint pain, and strong preference for denecimig over previous treatment. Results were comparable between denecimig QW and QM dosing frequencies, further supporting the flexibility of the tiered dosing approach for denecimig prophylaxis.

Efficacy outcomes with denecimig in adolescents and adults over 52 weeks in FRONTIER2 were comparable with the 26-week main phase [17], as well as with pediatric patients (aged <12 years) in the phase 3 FRONTIER3 study [20], demonstrating consistency of denecimig for maintaining bleed prevention over time and across age groups. Although direct comparisons cannot be made in the absence of head-to-head studies, ABR values achieved with denecimig during FRONTIER2 are comparable to ABR outcomes observed with ultra-extended half-life molecules, FVIIIa mimetics, and rebalancing agents for HA [5,[21], [22], [23]].

As observed with 26-week data from FRONTIER2 [17], numerically lower ABR values and a higher proportion of patients with zero bleeds were reported in the prestudy on-demand treatment group compared with the prestudy CFC prophylaxis group. One potential hypothesis regarding differences in ABR between the prestudy on-demand and CFC prophylaxis groups is that these differences may, in part, be influenced by historical experience with bleed treatment. Patients previously receiving on-demand treatment may be less familiar with assessing or treating a bleed themselves and may be hesitant to pursue a hospital visit due to historical experience or practical reasons. In addition, the ability of patients to effectively identify and distinguish true bleeding events can impact interpretation of efficacy outcomes. Imaging-based objective joint assessment tools were not included in the present study but should be considered in future trials of hemophilia treatments to more effectively identify incidence of true bleeding events.

Denecimig has been optimized to deliver high cofactor activity for FX activation [1]. In an *ex vivo* analysis, denecimig demonstrated ∼15-fold greater potency than emicizumab for thrombin generation [15]. However, a head-to-head trial with emicizumab has not yet been conducted to demonstrate if the pharmacodynamic superiority observed in *ex vivo* assays would translate into improved clinical outcomes.

The present study reports the first data assessing the effect of denecimig prophylaxis on joint symptoms. By the end of the 52-week treatment period, the majority of patients with baseline target joints had all target joints resolved. These data were supported by low estimated mean ABRs for joint bleeds, as well as some reduction in joint pain (assessed by the JPRS).

Across 52 weeks, no thrombotic events, hypersensitivity reactions, or clinical evidence of neutralizing antibodies were reported with denecimig prophylaxis, and safety findings showed no pattern or substantial differences by dosing frequency. Consistent with the FRONTIER2 26-week main phase [17], there was a low rate of ISRs (<2.2% of injections across each treatment arm) reported across the study period; the majority of these were transient, all were mild, and all recovered/resolved. Potential reasons for a low rate of ISRs with denecimig prophylaxis may in part be attributed to the low 0.8 mL injection volume and well-tolerated drug formulation and excipients [[24], [25], [26]].

Over 52 weeks, participants treated with denecimig experienced a reduction in joint pain (as measured by JPRS) and improvements in pain and physical functioning across several PRO measures in FRONTIER2, indicating the potential for denecimig to improve physical health status beyond bleeding outcomes. The majority of patients expressed a very strong or fairly strong preference for denecimig over their previous on-demand or CFC prophylaxis treatment, as measured by the HPPQ. The HPPQ has not yet been validated as a measurement tool; however, the favorable patient preference for denecimig suggests the potential for improving patient experience compared with standard of care. This may in part be attributed to the reported reduction in treatment burden, as measured by the Hemo-TEM, which was considered clinically meaningful (score improvement ≥8 points) [18] in a high proportion of study participants receiving denecimig.

Reductions in treatment burden with denecimig may in part be due to subcutaneous administration and reduced dosing frequency compared with conventional factor replacement. Furthermore, tiered dosing is designed to reduce the burden of administration by removing the need for complex dose calculations and adjustments based on weight [16]. Preference for denecimig over previous treatment in FRONTIER2 is supported by the denecimig device handling study, whereby the majority of patients demonstrated a preference for denecimig administered using a pen injector over previous emicizumab treatment [27].

FRONTIER2 used a randomized, controlled design and large cohort covering a broad population of people with HA, including adolescents and adults with HA of any disease severity, positive or negative inhibitor status, and both males and females. The FRONTIER4 (NCT05685238) long-term extension study is designed to assess denecimig outcomes beyond 1 year.

Limitations of the study include the lack of a comparator during the extension phase, which may affect the interpretation and impact of the PRO results, and therefore preclude causal inferences between denecimig use and improvements in PROs due to confounding factors such as regression toward the mean and treatment expectations. Bleeding episodes were self-reported, and therefore patients may potentially misclassify pain related to arthropathy or joint health as pain related to bleeds, which can inflate bleed reporting in patients with established arthropathy [28]. Imaging-based joint endpoints were not included in this study, and as such, a level of subjectivity remains; future assessments of hemophilia treatments should consider incorporation of validated ultrasound-based or magnetic resonance imaging-based scoring to objectively verify bleeds and monitor changes in joint health. Given the low number of patients in the QW and QM groups of arm 1 during the extension phase, and the relatively wide 95% CI for the estimated mean ABR, the statistical precision for the ABR outcomes for those groups is limited. The HPPQ tool used to report treatment preference is bespoke to the context of hemophilia and not yet validated at the time of writing.

## 5 Conclusions

During the FRONTIER2 main and extension phase, denecimig prophylaxis provided sustained bleed protection over 52 weeks in adolescents and adults with HA, irrespective of inhibitor status or disease severity, and was well tolerated. A high proportion of patients achieved zero treated bleeds, and denecimig maintained low ABRs across QW and QM dosing frequencies. PROs demonstrated that denecimig reduced treatment burden, improved physical function, reduced joint pain, and was strongly preferred over prior treatment. Together, these findings support use of denecimig as a flexible, subcutaneous prophylaxis intervention with the potential to reduce treatment burden while maintaining efficacy and safety over 1 year. The FRONTIER4 study will evaluate long-term safety and efficacy of denecimig.
