# Supplementary material for: Denecimig (Mim8) prophylaxis over 52 weeks in adolescents and adults with hemophilia A: FRONTIER2 extension study
Source: Res Pract Thromb Haemost. 2026 Aug 27;10(6):106915. doi: 10.1016/j.rpth.2026.106915 (PMC13627112; doi:10.1016/j.rpth.2026.106915)
Supplement: Supplementary file 1 [file mmc1.docx]

**Denecimig (Mim8) prophylaxis over 52 weeks in adolescents and adults with haemophilia A: FRONTIER2 extension study**

**Supplementary materials**

Victor Jiménez-Yuste, Aby Abraham, Ciprian Tomuleasa, Hazza Alzahrani, Lize van Vulpen, Renchi Yang, Samya Obaji, Sanjay Ahuja, Sophie Susen, Steven R. Lentz, Amalie Roar Høgh, Ilgiz Rakhmatullin, Cédric Hermans

# Supplementary Methods

**Negative binomial regression model**

The negative binomial regression models used for assessing the number of treated bleeds and bleed subtypes varied by treatment arm:

Number of treated bleeds:

- Arm 1: Logarithm of exposure time as offset, treatment as factor, and historical bleeding frequency (during the 12 months prior to enrolment) as covariate
- Arms 2a and 2b: Logarithm of exposure time as offset, treatment, inhibitor status and historical bleeding frequency (annualised bleeding rate [ABR] <18 or ABR ≥18) as factors, and historical bleeding frequency (during the 12 months prior to enrolment) as covariate
- Arms 3 and 4: Logarithm of exposure time as offset, severity of haemophilia (<1% or ≥1% factor VIII [FVIII] activity) as factor, and within-participant repeated measurements incorporated using an unstructured covariance structure

Bleed subtypes (treated spontaneous, treated traumatic, treated joint and treated target joint bleeds):

- Arm 1: No analyses were conducted
- Arms 2a and 2b: Model as above
- Arms 3 and 4: Logarithm of exposure time was used as an offset, severity of haemophilia (<1% or ≥1% FVIII activity) as factor and historical ABR as a covariate. The historical ABR was the ABR for treated bleeds during the run-in period (for patients from mainland China, the historical ABR was based on the treated bleeds the year prior to enrolment

## Supplementary Figures

Supplementary Figure 1. FRONTIER2 study design

Patients who completed the extension had the option to transfer to the OLE study FRONTIER4 (NCT05685238). Patients who discontinued or did not wish to enter the extension completed a follow-up visit 21 weeks after the last dose was administered. This study included 27 patients from mainland China who were not included in the FRONTIER2 26-week primary analysis (Mancuso ME et al. *N Engl J Med* 2026;30;394:1696–709).
*Patients in the pre-study on-demand treatment group were stratified by factor VIII inhibitor status and ABR (<18 or ≥18). ^†^Patients in the pre-study CFC prophylaxis group were stratified by haemophilia severity.
CFC, clotting factor concentrate; HA, haemophilia A; HAwI, haemophilia A with inhibitors; OLE, open-label extension; QM, once-every-month; QW, once-every-week.

Supplementary Figure 2. Participant disposition across the main and extension phase of FRONTIER2

AE, adverse event; OD, on-demand; QM, once-every-month; QW, once-every-week.

**Supplementary Figure 3. Laboratory assessments of FRONTIER2: (A) D-Dimer, (B) prothrombin fragments 1 + 2, (C) FIX antigen, (D) FX antigen, (E) fibrinogen**

A) D-Dimer

B) Prothrombin fragments 1 + 2
C) FIX antigen
D) FX antigen

E) Fibrinogen

The patients in Arm 1 start denecimig treatment in the extension period. Outlier values not included in the figures (D-dimer): Subject 272001 in Arm 2a at Day 56 had a value of 3651 µg/L DDU. Outlier values not included in the figures (prothrombin fragment 1 and 2): Subject 271203 in Arm 1 at Day 91 had a value of 5480 pmol/L. Subject 272001 in Arm 2a at Day 146 and day 406 had a value of 12,000 pmol/L. Subject 382001 in Arm 2a at Day 380 had a value of 9090 pmol/L. Subject 501204 in Arm 2a at Day 181 had a value of 8090 pmol/L. Subject 501206 in Arm 2a at Day 356 had a value of 4300 pmol/L. Subject 501202 in Arm 2b at Day 0 had a value of 11,860 pmol/L. Subject 862206 in Arm 2b at Day 359 had a value of 9350 pmol/L. Subject 117203 in Arm 3 at Day 56 had a value of 8360 pmol/L. Subject 332203 in Arm 3 at Day 27 had a value of 12,000 pmol/L. Subject 982202 in Arm 3 at Day 363 had a value of 11,930 pmol/L. Subject 811201 in Arm 4 at Day 27 had a value of 12,000 pmol/L. Subject 862204 in Arm 4 at Day 359 had a value of 12,000 pmol/L.
DDU, D-dimer units; FIX, factor IX; FX, factor X; QW, once-weekly; QM, once-monthly.

## Tables

**Supplementary Table 1. Treated bleeding episodes during the main phase (Weeks 0–26) of FRONTIER2.**

|  | Pre-study on-demand treatment group | | | Pre-study CFC prophylaxis group | |
| --- | --- | --- | --- | --- | --- |
|  | On demand (Arm 1) | Denecimig QW  (Arm 2a) | Denecimig QM  (Arm 2b) | Denecimig QW  (Arm 3) | Denecimig QM  (Arm 4) |
|  | *n* = 18 | *n* = 22 | *n* = 21 | *n* = 111 | *n* = 109 |
| Mean exposure time, years | 0.50 | 0.47 | 0.50 | 0.48 | 0.50 |
| Number of patients with bleeds,  *n* (%) | 18 (100.0) | 3 (13.6) | 2 (9.5) | 37 (33.3) | 40 (36.7) |
| Number of patients with zero bleeds, *n* (%) | 0 (0.0) | 19 (86.4) | 19 (90.5) | 74 (66.7) | 69 (63.3) |
| Number of treated bleeds | 158 | 6 | 4 | 107 | 97 |
| ABR |  |  |  |  |  |
| Estimated mean [95% CI] | 16.09  [11.21;23.09] | 0.43  [0.17;1.07] | 0.25  [0.08;0.76] | 2.32  [1.35;3.99] | 1.79  [1.22;2.63] |
| Median [IQR] | 12.24  [8.03;20.75] | 0.00  [0.00;0.00] | 0.00  [0.00;0.00] | 0.00  [0.00;2.01] | 0.00  [0.00;2.01] |

This analysis included 27 patients from mainland China who were not included in the FRONTIER2 26-week primary analysis (Mancuso ME et al. *N Engl J Med* 2026;30;394:1696–709).
ABR, annualised bleeding rate; CFC, clotting factor concentrate; CI, confidence interval; IQR, interquartile range; QM, once-every-month; QW, once-every-week.

**Supplementary Table 2. Treated bleeding episodes during the extension phase (Weeks 26–52) of FRONTIER2.**

|  | Pre-study on-demand treatment group | | | | Pre-study CFC prophylaxis group | |
| --- | --- | --- | --- | --- | --- | --- |
|  | Denecimig QW (Arm 1) | Denecimig QM  (Arm 1) | Denecimig QW  (Arm 2a) | Denecimig QM  (Arm 2b) | Denecimig QW  (Arm 3) | Denecimig QM  (Arm 4) |
|  | *n* = 8 | *n* = 10 | *n* = 21 | *n* = 21 | *n* = 104 | *n* = 108 |
| Mean exposure time, years | 0.51 | 0.51 | 0.52 | 0.51 | 0.51 | 0.50 |
| Number of patients with bleeds,  *n* (%) | 1 (12.5) | 3 (30.0) | 2 (9.5) | 3 (14.3) | 31 (29.8) | 34 (31.5) |
| Number of patients with zero bleeds, *n* (%) | 7 (87.5) | 7 (70.0) | 19 (90.5) | 18 (85.7) | 73 (70.2) | 74 (68.5) |
| Number of treated bleeds | 3 | 4 | 7 | 4 | 70 | 84 |
| ABR |  |  |  |  |  |  |
| Estimated mean [95% CI] | 0.67  [0.13;3.61] | 0.79  [0.19;3.33] | 0.45  [0.19;1.08] | 0.25  [0.08;0.77] | 1.28  [0.78;2.08] | 1.54  [0.92;2.59] |
| Median [IQR] | 0.00  [0.00;0.00] | 0.00  [0.00;1.83] | 0.00  [0.00;0.00] | 0.00  [0.00;0.00] | 0.00  [0.00;1.92] | 0.00  [0.00;1.93] |

ABR, annualised bleeding rate; CFC, clotting factor concentrate; CI, confidence interval; IQR, interquartile range; QM, once-every-month; QW, once-every-week.

Supplementary Table 3. Treated bleeding episodes by inhibitor status at baseline in the pre-study on-demand treatment group (Arms 2a and 2b)

|  | Pre-study on-demand treatment group: Denecimig QW (Arm 2a) and QM (Arm 2b)  Main and Extension | |
| --- | --- | --- |
|  | Inhibitor negative | Inhibitor positive |
|  | *n* = 24 | *n* = 19 |
| Mean treatment analysis period (years) | 0.97 | 1.01 |
| Treated bleeding episodes | | |
| Patients with zero treated bleeds, % [95% CI] | 83.3 [62.6;95.3] | 89.5 [66.9;98.7] |
| Number of treated bleeds | 19 | 2 |
| Estimated mean ABR [95% CI] | 0.58 [0.30;1.10] | 0.09 [0.02;0.39] |
| Median ABR [IQR] | 0.00 [0.00;0.00] | 0.00 [0.00;0.00] |

The negative binomial model included historical bleeding frequency (ABR <18 or ABR ≥18) as a factor, historical bleeding frequency as a covariate and an interaction term between treatment and inhibitor status, and the logarithm of treatment duration was used as an offset.
ABR, annualised bleeding rate; CI, confidence interval; IQR, interquartile range; QM, once-every-month; QW, once-every-week.

Supplementary Table 4. Treated bleeding episodes by haemophilia severity at baseline in the pre-study CFC prophylaxis group (Arms 3 and 4)

|  | Pre-study CFC prophylaxis group: Denecimig QW (Arm 3) and QM (Arm 4)  Main and Extension | |
| --- | --- | --- |
|  | Non-severe haemophilia | Severe haemophilia |
|  | *n* = 30 | *n* = 190 |
| Mean treatment analysis period (years) | 0.97 | 0.97 |
| Treated bleeding episodes | | |
| Patients with zero treated bleeds, % [95% CI] | 63.3 [43.9;80.1] | 47.4 [40.1;54.7] |
| Number of bleeds | 50 | 308 |
| Estimated mean ABR [95% CI] | 1.47 [0.78;2.79] | 1.66 [1.28;2.16] |
| Median ABR [IQR] | 0.00 [0.00;1.00] | 0.97 [0.00;1.97] |

The negative binomial model included an interaction term between treatment and haemophilia severity (≥1% or <1% endogenous factor VIII), historical ABR is included as a covariate, and the logarithm of treatment duration was used as an offset. The historical ABR was the ABR for treated bleeds during the run-in period, and for patients from mainland China, the historical ABR was based on the treated bleeds the year prior to enrolment.
ABR, annualised bleeding rate; CFC, clotting factor concentrate; CI, confidence interval; IQR, interquartile range; QM, once-every-month; QW, once-every week.

Supplementary Table 5. Treated bleeding episodes by subtype of bleed during denecimig prophylaxis

|  | Pre-study on-demand treatment group | | Pre-study CFC prophylaxis group | | |
| --- | --- | --- | --- | --- | --- |
|  | Denecimig QW  (Arm 2a) | Denecimig QM  (Arm 2b) | | Denecimig QW  (Arm 3) | Denecimig QM  (Arm 4) |
|  | Main and Extension | Main and Extension | | Main and Extension | Main and Extension |
|  | *n* = 22 | *n* = 21 | | *n* = 111 | *n* = 109 |
| Mean treatment analysis period, years | 0.97 | 1.01 | | 0.96 | 0.99 |
| Treated spontaneous bleeding episodes |  |  | |  |  |
| Patients with zero bleeds, % [95% CI] | 90.9 [70.8;98.9] | 85.7 [63.7;97.0] | | 73.0 [63.7;81.0] | 76.1 [67.0;83.8] |
| Number of bleeds | 2 | 7 | | 93 | 86 |
| Median ABR | 0.00 | 0.00 | | 0.00 | 0.00 |
| Estimated mean ABR [95% CI] | 0.08 [0.02;0.35] | 0.25 [0.10;0.62] | | 1.50 [0.81;2.75] | 0.57 [0.31;1.04] |
| Treated traumatic bleeding episodes |  |  | |  |  |
| Patients with zero bleeds, % [95% CI] | 90.9 [70.8;98.9] | 95.2 [76.2;99.9] | | 61.3 [51.5;70.4] | 59.6 [49.8;68.9] |
| Number of bleeds | 11 | 1 | | 78 | 85 |
| Median ABR | 0.00 | 0.00 | | 0.00 | 0.00 |
| Estimated mean ABR [95% CI] | 0.13 [0.03;0.46] | 0.01 [0.00;0.11] | | 0.63 [0.45;0.86] | 0.67 [0.50;0.92] |
| Treated joint bleeding episodes |  |  | |  |  |
| Patients with zero bleeds, % [95% CI] | 86.4 [65.1;97.1] | 90.5 [69.6;98.8] | | 62.2 [52.5;71.2] | 67.0 [57.3;75.7] |
| Number of bleeds | 11 | 2 | | 105 | 116 |
| Median ABR | 0.00 | 0.00 | | 0.00 | 0.00 |
| Estimated mean ABR [95% CI] | 0.36 [0.16;0.86] | 0.07 [0.02;0.34] | | 1.27 [0.80;2.03] | 0.76 [0.48;1.18] |
| Treated target joint bleeding episodes* |  |  | |  |  |
| Patients with zero bleeds, % [95% CI] | 86.4 [65.1;97.1] | 100.0 [83.9;100.0] | | 91.0 [84.1;95.6] | 97.2 [92.2;99.4] |
| Number of bleeds | 5 | 0 | | 26 | 32 |
| Median ABR | 0.00 | 0.00 | | 0.00 | 0.00 |
| Estimated mean ABR [95% CI] | 0.18 [0.06;0.56] | - | | 0.06 [0.02;0.20] | 0.06 [0.02;0.19] |

*A target joint is defined as a joint in which 3 or more spontaneous bleeding episodes have occurred within 6 months before the date of the assessment. When there have been ≤2 bleeds into the joint within a consecutive 12-month period, the joint is no longer considered a target joint; 12-month data are needed to check for target joint resolution; Arm 1 is not included as patients cannot be checked for resolution of baseline target joints, due to having less than 12 months of denecimig treatment.
For Arms 2a and 2b, the negative binomial model includes treatment, inhibitor status and historical bleeding frequency (ABR <18 or ABR ≥18) as factors and historical bleeding frequency as covariate and the logarithm of treatment duration is used as an offset. For Arms 3 and 4, the negative binomial model includes treatment and severity of haemophilia (≥1% or <1% endogenous factor VIII) as factors, historical ABR is included as a covariate, and the logarithm of treatment duration is used as an offset. The historical ABR is the ABR for treated bleeds during the run-in period, and for the Chinese patients the historical ABR is based on the treated bleeds the year prior to enrolment. Analysis by subtype of bleeds was not conducted for Arm 1 extension due to sparse bleed data.
ABR, annualised bleeding rate; CFC, clotting factor concentrate; CI, confidence interval; QM, once-every-month; QW, once-every-week.

Supplementary Table 6. Number of injections to treat breakthrough bleeds during denecimig prophylaxis

|  | Pre-study on-demand treatment group | | | | Pre-study CFC prophylaxis group | |
| --- | --- | --- | --- | --- | --- | --- |
|  | Denecimig QW  (Arm 1) | Denecimig QM  (Arm 1) | Denecimig QW  (Arm 2a) | Denecimig QM  (Arm 2b) | Denecimig QW  (Arm 3) | Denecimig QM  (Arm 4) |
|  | Extension | Extension | Main and Extension | Main and Extension | Main and Extension | Main and Extension |
|  | *n* = 8 | *n* = 10 | *n* = 22 | *n* = 21 | *n* = 111 | *n* = 109 |
| Number of patients, *n* (%) | | | | | | |
| with treated bleeds | 1 (12.5) | 3 (30.0) | 3 (13.6) | 3 (14.3) | 55 (49.5) | 56 (51.4) |
| with FVIII | 0 | 3 (30.0) | 2 (9.1) | 2 (9.5) | 54 (48.6) | 56 (51.4) |
| with bypassing agents | 1 (12.5) | 0 | 1 (4.5) | 0 | 1 (0.9) | 0 |
| with aPCC/PCC | 0 | 0 | 0 | 1 (4.8) | 0 | 0 |
| Number of treated bleeds | 3 | 4 | 13 | 8 | 177 | 181 |
| Total number of injections to treat the bleed, *n* (%)* | | | | | | |
| 1 injection | 3 (100) | 3 (75.0) | 11 (84.6) | 8 (100) | 117 (66.1) | 133 (73.5) |
| 2 injections | 0 | 0 | 1 (7.7) | 0 | 31 (17.5) | 27 (14.9) |
| ≥3 injections | 0 | 1 (25.0) | 1 (7.7) | 0 | 29 (16.4) | 21 (11.6) |
| Mean (SD) | 1.0 (0.0) | 1.5 (1.0) | 1.4 (1.1) | 1.0 (0.0) | 1.7 (1.5) | 1.6 (1.8) |
| Median | 1.0 | 1.0 | 1.0 | 1.0 | 1.0 | 1.0 |
| Min ; Max | 1.0 ; 1.0 | 1.0 ; 3.0 | 1.0 ; 5.0 | 1.0; 1.0 | 1.0; 11.0 | 1.0; 22.0 |

*Percentage represents the proportion of bleeds.
FVIII includes standard recombinant factor concentrate, extended half-life factor concentrate, and plasma-derived FVIII. Bypassing agents include recombinant activated factor II and activated factor VII + factor X.
aPCC, activated prothrombin complex concentrate; FVIII, factor VIII; PCC, prothrombin complex concentrate; QM, once-every-month; QW, once-every-week; SD, standard deviation.

Supplementary Table 7. Target joints and target joint resolution at the end of denecimig prophylaxis

|  | Pre-study on-demand treatment group | | Pre-study CFC prophylaxis group | |
| --- | --- | --- | --- | --- |
|  | Denecimig QW  (Arm 2a) | Denecimig QM  (Arm 2b) | Denecimig QW  (Arm 3) | Denecimig QM  (Arm 4) |
|  | Main and Extension | Main and Extension | Main and Extension | Main and Extension |
|  | *n* = 22 | *n* = 21 | *n* = 111 | *n* = 109 |
| Patients with target joints at baseline, *n* | 17 | 13 | 20 | 13 |
| Patients with all baseline target joints resolved at the end of denecimig treatment, *n* | 16 (94.1) | 13 (100.0) | 17 (85.0) | 10 (79.9) |
| Patients with one or more baseline target resolved at the end of denecimig treatment, *n* | 16 (94.1) | 13 (100.0) | 19 (95.0) | 11 (84.6) |
| Patients with target joints at baseline where resolution was evaluable due to adequate treatment time, *n* | 16* | 13 | 20 | 13 |
| Patients with all baseline target joints resolved at the end of denecimig treatment, *n* | 16 (100.0) | 13 (100.0) | 17 (85.0) | 10 (79.9) |
| Patients with one or more baseline target resolved at the end of denecimig treatment, *n* | 16 (100.0) | 13 (100.0) | 19 (95.0) | 11 (84.6) |
| Number of baseline target joints | 26 | 28 | 24 | 24 |
| Target joints resolved at the end of denecimig treatment, *n* | 23 (88.5) | 28 (100.0) | 21 (87.5) | 19 (79.2) |
| Number of baseline target joints where resolution was evaluable due to adequate treatment time | 23* | 28 | 24 | 24 |
| Target joints resolved at the end of denecimig treatment, *n* | 23 (100.0) | 28 (100.0) | 21 (87.5) | 19 (79.2) |
| Number of patients without target joints at randomisation with a new target joint at the end of denecimig treatment, *n* | 0 | 0 | 2 | 2 |
| Patients with new target joints at the end of denecimig treatment | 0 | 0 | 2 | 2 |
| 1 target joint | 0 | 0 | 2 | 0 |
| 2 target joints | 0 | 0 | 0 | 2 |
| ≥3 target joints | 0 | 0 | 0 | 0 |

*In Arm 2a, 1 subject had 3 baseline target joints, and as the treatment duration was 21 days, the resolution of baseline target joints could not be checked.
A target joint is defined as a joint in which 3 or more spontaneous bleeding episodes have occurred within 6 months before the date of the assessment. When there have been ≤2 bleeds into the joint within a consecutive 12-month period, the joint is no longer considered a target joint; 12-month data are needed to check for target joint resolution; Arm 1 is not included as patients cannot be checked for resolution of baseline target joints, due to having less than 12 months of denecimig treatment.
CFC, clotting factor concentrate; QM, once-every-month; QW, once-every-week.

Supplementary Table 8. Serious treatment-emergent adverse events by system organ class and preferred term (pre-study on-demand treatment group)

|  | Pre-study on-demand treatment group | | | |
| --- | --- | --- | --- | --- |
|  | Denecimig QW (Arm 1) | Denecimig QM  (Arm 1) | Denecimig QW  (Arm 2a) | Denecimig QM  (Arm 2b) |
|  | Extension | Extension | Main and Extension | Main and Extension |
|  | *n* = 8 | *n* = 10 | *n* = 22 | *n* = 21 |
| Total exposure time (years) | 4.09 | 5.17 | 21.65 | 21.23 |
| All serious TEAEs, *n* (%) E [R] | 0 | 0 | 1 (4.5) 2 [ 0.09] | 0 |
| Infections and infestations | 0 | 0 | 1 (4.5) 1 [ 0.05] | 0 |
| Psoas abscess | 0 | 0 | 1 (4.5) 1 [ 0.05] | 0 |
| Nervous system disorders | 0 | 0 | 1 (4.5) 1 [ 0.05] | 0 |
| VI^th^ nerve paralysis* | 0 | 0 | 1 (4.5) 1 [ 0.05] | 0 |

*Event considered probably related to treatment by investigator.
All TEAEs in the on-treatment period are included in this output, including those in the follow-up period.
CFC, clotting factor concentrate; E, number of adverse events; QM, once-every-month; QW, once-every-week; R, rate (number of adverse events per participant-year of exposure); TEAE, treatment-emergent adverse event.

Supplementary Table 9. Serious treatment-emergent adverse events by system organ class and preferred term (pre-study CFC prophylaxis group)

|  | Pre-study CFC prophylaxis group | |
| --- | --- | --- |
|  | Denecimig QW (Arm 3) | Denecimig QM (Arm 4) |
|  | Main and Extension | Main and Extension |
|  | *n* = 111 | *n* = 109 |
| Total exposure time (years) | 109.95 | 109.85 |
| All serious TEAEs, *n* (%) E [R] | 7 (6.3) 9 [0.08] | 9 (8.3) 12 [0.11] |
| Infections and infestations | 2 (1.8) 2 [0.02] | 4 (3.7) 6 [0.05] |
| Appendicitis | 1 (0.9) 1 [0.01] | 1 (0.9) 1 [0.01] |
| Bronchitis | 0 | 1 (0.9) 2 [0.02] |
| Abdominal abscess | 0 | 1 (0.9) 1 [0.01] |
| Cellulitis | 0 | 1 (0.9) 1 [0.01] |
| Pneumonia | 0 | 1 (0.9) 1 [0.01] |
| Soft tissue infection | 1 (0.9) 1 [0.01] |  |
| Injury, poisoning and procedural complications | 1 (0.9) 2 [0.02] | 1 (0.9) 2 [0.02] |
| Head injury | 0 | 1 (0.9) 1 [0.01] |
| Ligament sprain | 1 (0.9) 1 [0.01] | 0 |
| Skin laceration | 1 (0.9) 1 [0.01] | 0 |
| Limb injury | 0 | 1 (0.9) 1 [0.01] |
| Gastrointestinal disorders | 1 (0.9) 1 [0.01] | 1 (0.9) 1 [0.01] |
| Haemorrhoids | 0 | 1 (0.9) 1 [0.01] |
| Upper gastrointestinal haemorrhage | 1 (0.9) 1 [0.01] | 0 |
| Musculoskeletal and connective tissue disorders | 0 | 2 (1.8) 2 [0.02] |
| Arthropathy | 0 | 1 (0.9) 1 [0.01] |
| Synovitis | 0 | 1 (0.9) 1 [0.01] |
| Eye disorders | 1 (0.9) 1 [0.01] | 0 |
| Optic ischaemic neuropathy | 1 (0.9) 1 [0.01] | 0 |
| Hepatobiliary disorders | 1 (0.9) 1 [0.01] | 0 |
| Cholecystitis chronic | 1 (0.9) 1 [0.01] | 0 |
| Neoplasms benign, malignant and unspecified | 1 (0.9) 1 [0.01] | 0 |
| Testicular teratoma benign | 1 (0.9) 1 [0.01] | 0 |
| Nervous system disorders | 0 | 1 (0.9) 1 [0.01] |
| Epilepsy | 0 | 1 (0.9) 1 [0.01] |
| Product issues | 1 (0.9) 1 [0.01] | 0 |
| Device loosening | 1 (0.9) 1 [0.01] | 0 |

All TEAEs in the on-treatment period are included in this output including the ones in follow-up period.
CFC, clotting factor concentrate; E, number of adverse events; QM, once-every-month; QW, once-every-week; R, rate (number of adverse events per participant-year of exposure); TEAE, treatment-emergent adverse event.

Supplementary Table 10. Injection-site reactions during denecimig prophylaxis

|  | Pre-study on-demand treatment group | | | | Pre-study CFC prophylaxis group | |
| --- | --- | --- | --- | --- | --- | --- |
|  | Denecimig QW (Arm 1) | Denecimig QM  (Arm 1) | Denecimig QW  (Arm 2a) | Denecimig QM  (Arm 2b) | Denecimig QW  (Arm 3) | Denecimig QM  (Arm 4) |
|  | Extension | Extension | Main and Extension | Main and Extension | Main and Extension | Main and Extension |
|  | *n* = 8 | *n* = 10 | *n* = 22 | *n* = 21 | *n* = 111 | *n* = 109 |
| Total exposure time (years) | 4.09 | 5.17 | 21.65 | 21.23 | 109.95 | 109.85 |
| Number of injections | 220 | 72 | 1132 | 280 | 5661 | 1437 |
| Number of injection-site reactions | 1 | 0 | 2 | 2 | 124 | 22 |
| Percent of injections leading to injection-site reaction | 0.45 | 0 | 0.18 | 0.71 | 2.19 | 1.53 |
| Number of patients with injection-site reactions | 1 | 0 | 2 | 1 | 14 | 11 |
| Percent of patients with injection-site reactions | 12.5 | 0 | 9.1 | 4.8 | 12.6 | 10.1 |
| Rate of injection-site reactions per participant year of exposure | 0.24 | 0 | 0.09 | 0.09 | 1.13 | 0.20 |

CFC, clotting factor concentrate; QM, once-every-month; QW, once-every-week.

Supplementary Table 11. Anti-denecimig antibodies during denecimig prophylaxis

|  | Pre-study on-demand treatment group | | | | Pre-study CFC prophylaxis group | | Total |
| --- | --- | --- | --- | --- | --- | --- | --- |
|  | Denecimig QW  (Arm 1) | Denecimig QM  (Arm 1) | Denecimig QW  (Arm 2a) | Denecimig QM  (Arm 2b) | Denecimig QW  (Arm 3) | Denecimig QM  (Arm 4) |  |
|  | Extension | Extension | Main and Extension | Main and Extension | Main and Extension | Main and Extension |  |
|  | *n* = 8 | *n* = 10 | *n* = 22 | *n* = 21 | *n* = 111 | *n* = 109 | *N* = 281 |
| Number of patients with anti-denecimig antibodies, *n* (%) | 0 | 0 | 2 (9.1) | 2 (9.5) | 12 (10.8) | 5 (4.6) | 21 (7.5) |
| Treatment emergent | 0 | 0 | 2 (9.1) | 2 (9.5) | 10 (9.0) | 5 (4.6) | 19 (6.8) |
| Pre-existing | 0 | 0 | 0 | 0 | 2 (1.8) | 0 | 2 (0.7) |
| Titre evaluation, *n* (%) |  | | | | | | |
| Low titre | 0 | 0 | 2 (9.1) | 2 (9.5) | 11 (9.9) | 5 (4.6) | 20 (7.1) |
| Medium titre | 0 | 0 | 0 | 0 | 1 (0.9) | 0 | 1 (0.4) |
| High titre | 0 | 0 | 0 | 0 | 0 | 0 | 0 |

*Percentages for ADAs by titre categories are expressed as a proportion the total patients per cohort.
Low titre: below regulatory detection threshold of 100 ng/mL ADA (titre 33–2112). Medium titre: 100–800 ng/mL ADA (titre 4224–16,896). High titre: Above 800 ng/mL ADA (titre ≥33,792).
ADA, anti-drug antibody; CFC, clotting factor concentrate; QM, once-every-month; QW, once-every-week.

Supplementary Table 12. Patient-reported outcomes during the main and extension phases of FRONTIER2

|  | Pre-study on-demand treatment group | | Pre-study CFC prophylaxis group |
| --- | --- | --- | --- |
|  | Denecimig QW/QM (Arm 1) | Denecimig QW/QM  (Arms 2a and 2b) | Denecimig QW/QM  (Arms 3 and 4) |
|  | Extension | Main and Extension | Main and Extension |
| Hemo-TEM Total Score | | | |
| Number of patients, *n* | 13 | 29 | 182 |
| Mean baseline score (SD) | 24.1 (16.8) | 16.9 (13.7) | 14.7 (12.6) |
| Change from baseline to Week 52 (SD) | –16.0 (16.1) | –12.7 (12.7) | –9.7 (11.6) |
| Patients with clinically meaningful* improvement in score at Week 52, *n* (%) | 8 (61.5) | 16 (55.2) | 84 (46.2) |
| HPPQ: Treatment preference by proportion of patients at Week 52 | | | |
| Number of patients, *n* | 17 | 40 | 192 |
| Preference for denecimig, *n* (%) | 16 (94.1) | 40 (100.0) | 178 (92.7) |
| Very strong preference, *n* (%) | 11 (64.7) | 26 (65.0) | 134 (69.8) |
| Fairly strong, *n* (%) | 4 (23.5) | 13 (32.5) | 41 (21.4) |
| Not very strong, *n* (%) | 1 (5.9) | 1 (2.5) | 3 (1.6) |
| Preference for previous treatment, *n* (%) | 0 | 0 | 6 (3.1) |
| Very strong preference, *n* (%) | 0 | 0 | 3 (1.6) |
| Fairly strong, *n* (%) | 0 | 0 | 2 (1.0) |
| Not very strong, *n* (%) | 0 | 0 | 1 (0.5) |
| No preference, *n* (%) | 1 (5.9) | 0 | 8 (4.2) |
| PedsQL™ physical functioning | | | |
| Number of patients, *n* | 13 | 29 | 167 |
| Mean baseline score (SD) | 50.0 (26.8) | 59.7 (23.6) | 74.4 (20.3) |
| Change from baseline to Week 52 (SD) | 13.0 (16.5) | 17.6 (20.8) | 4.0 (12.4) |
| PGI-C change in physical function at Week 52 | | | |
| Number of patients, *n* | 17 | 40 | 192 |
| Much better, *n* (%) | 10 (58.8) | 26 (65.0) | 77 (40.1) |
| A little better, *n* (%) | 7 (41.2) | 10 (25.0) | 61 (31.8) |
| No change, *n* (%) | 0 | 3 (7.5) | 52 (27.1) |
| A little worse, *n* (%) | 0 | 1 (2.5) | 2 (1.0) |
| Much worse, *n* (%) | 0 | 0 | 0 |
| PGI-C change in pain intensity at Week 52 | | | |
| Number of patients, *n* | 17 | 40 | 192 |
| Much less pain, *n* (%) | 11 (64.7) | 30 (75.0) | 72 (37.5) |
| A little less pain, *n* (%) | 4 (23.5) | 8 (20.0) | 43 (22.4) |
| No change, *n* (%) | 1 (5.9) | 2 (5.0) | 70 (36.5) |
| A little more pain, *n* (%) | 0 | 0 | 6 (3.1) |
| Much more pain, *n* (%) | 1 (5.9) | 0 | 1 (0.5) |
| JPRS | | | |
| Number of patients, *n* | 13 | 29 | 184 |
| Mean baseline score (SD) | 4.2 (2.9) | 3.3 (2.6) | 2.6 (2.6) |
| Change from baseline to Week 52 (SD) | –1.8 (2.5) | –1.5 (3.1) | –0.4 (2.0) |

*Clinically meaningful improvement in the Hemo-TEM total score defined as achieving a reduction of ≥8 points [19].
Hemo‑TEM: scale of 0–100, with higher scores reflecting greater burden. PedsQL™ physical functioning domain: scale of 0–100 with higher scores indicate better physical functioning. JPRS: scale of 0–10, with higher scores reflecting greater pain intensity. PGI‑C change in physical function: Patients responded to “How would you rate your current ability to do every day physical activities compared to your ability to do these activities before the study?” on a 5-item scale ranging from “Much worse” to “Much better”. PGI‑C change in pain intensity: Patients responded to “How much pain in your joints do you have now compared to before the study?” on a 5‑item scale ranging from “Much more pain” to “Much less pain”. HPPQ responded to “Overall what treatment do you prefer?” with response options “Current, “Previous” and “No preference”. If “Current” or “Previous” was selected, a follow-up question was asked “How strong is that preference?”, with patients responding “Very strong”, “Fairly strong” or “Not very strong”.
CFC, clotting factor concentrate; Hemo‑TEM, Hemophilia Treatment Experience Measure; HPPQ, Haemophilia Patient Preference Questionnaire; JPRS, Joint Pain Rating Scale; PedsQL™, Pediatric Quality of Life Inventory; PGI-C, Patient Global Impression of Change; QM, once-every-month; QW, once-every-week; SD, standard deviation.
